# Supplementary material for: Prevalence of enteric pathogens, intestinal parasites and resistance profile of bacterial isolates among HIV infected and non-infected diarrheic patients in Dessie Town, Northeast Ethiopia
Source: PLoS One. 2020 Dec 15;15(12):e0243479. doi: 10.1371/journal.pone.0243479 (PMC7737993; doi:10.1371/journal.pone.0243479)
Supplement: S1 Questionnaire — (DOCX) [file pone.0243479.s001.docx]

**Questionnaire (English Version)**

This questionnaire is prepared by Mr. Assefa Belay in Addis Ababa University, College of Health Sciences that aimed to determine the prevalence of *Salmonella* and *Shigella* and their antimicrobial susceptibility patterns among HIV infected and non-infected clients with acute diarrhea at Dessie selected health facilities, Northeast Ethiopia.

Date:___________ Participant ID:_______

**Questionnaire (English Version)**

| **Part I: Socio-demographic characteristics** | | | | |
| --- | --- | --- | --- | --- |
| Identification code ________________ | | | | |
| No. | Questionnaire on identification of the respondents | Alternative choice for  Responses | Skip | Code |
| 101 | Age | _____ year |  |  |
| 102 | Sex | A. Male  B. Female |  |  |
| 103 | Residence (From where did you come) | A. Urban  B. Rural |  |  |
| 104 | What is your occupation? | A. Government employee  B. Housewife  C. Merchant  D. Student  E. Other |  |  |
| 105 | What is your educational status? | A. Illiterate  B. Only read & write  C. Primary school completed  D. Secondary school completed  E. College or university |  |  |
| 106 | Monthly income in ETB? | A. ≤500  B. 501-1000  C. 1001-1500  D. 1501-2000  E. >2000 |  |  |
| **Part II: Clinical data** | | | | |
| 201 | Duration of diarrhea | Days _______ |  |  |
| 202 | 2. Consistency of diarrhea | A. Watery  B. Mucoid  C. Bloody  D. Mixed (Mucoid + blood) |  |  |
| 203 | History of HIV/AIDS | A. Yes  B. No  C. Not tested |  |  |
| 204 | If yes, recent CD4 count | Cells/uL |  |  |
| 205 | Vomiting: | A. Yes  B. No |  |  |
| 206 | Abdominal Cramp | A. Yes  B. No |  |  |
| **III. Risk factors** | | | | |
| 301 | Presence of latrine at home: | A. Yes  B. No |  |  |
| 302 | If “No” number 301, where do you defecate? | A. Public latrine  B. Open Field  C. River |  |  |
| 303 | Source of drinking water | A. Private Pipe water  B. Public pipe water  C. River water  D. Well water |  |  |
| 304 | Hand washing habit before meal: | A. Yes  B. No |  |  |
| 305 | If “Yes”, for number 304, do you use soap | A. With soap  B. Without soap |  |  |

**Part III: Laboratory data:**

Code: _________

**1. Stool Culture and identification:**

XLD culture result: Positive: _____________ Negative _______________

Isolated Bacteria ______________________

**2. Antimicrobial susceptibility testing**

**Antimicrobial disks S (mm) I (mm) R (mm)**

Amoxicillin (AML, 25µg) ------ ------ ------

Ampicillin (AMP, 10μg) ------ ------ -------

Ceftriaxone (CRO, 30µg). ------- ------- -------

Tetracycline (TTC 30-μg) ------- ------- -------

Chloramphenicol (CAF, 30-μg), ------- ------- -------

Ciprofloxacin (CIP,5-μg) ------- ------- -------

Trimethoprim-sulfamethoxazole (SXT, 25μg), ------- ------- -------

Comments: ___________________________________

**መጠይቅ (Amharic Version)**

ÃI SÖÃp ¾}²ÒË¨< ¾›Ç=e ›vv ¿”y`c=+Ã ¾Ö?“ dÃ”e }T] uJ’<ƒ u›„ ›có uLÃ ¾G<K}— Ç=Ó] KSS[mÁ êG<õ c=J” ›LT¨< ucT@” Ue^p uÅc? ›Ÿ”vu= }pTØ ›Uß ¾J’<ƒ” cMV’@L“ g=Ñ@L ¾T>vK< v¡ቴ]Á­‹” e`ß~” KT¨p“ KSÉH’>ƒ ÁK¨<” Óƒ`’ƒ Å[Í Td¾ƒ uT>M zÃ[c< uÅT†¨< vKv†¨<“ uK?Kv†¨< ui}™‹ ¾T>Å[Ó SÖÃp ’¨<፡፡

| ክፍል 1: የግለሰቡማህበራዊእናኢኮኖሚያዊመረጃ | | | | | |
| --- | --- | --- | --- | --- | --- |
| የተሳታፊው መለያ ቁጥር ________ | | | | | |
| ተ.ቁ | የግለሰቡ አካላዊ ማህበራዊ እና ኢኮኖሚያዊ መጠይቅ ዝርዝር | የመልስ አማራጮች | | ይለፍ | መለያ ቁጥር |
| 101 | እድሜ | _______ አመት | |  |  |
| 102 | ë} | ¨”É  c?ƒ | |  |  |
| 103 | **የመኖሪያአድራሻ** | ከተማ  ገጠር | |  |  |
| 104 | **ስራ?** | የመንግስትሰራተኛ  የቤትእመቤት  የግልስራ  4. ተማሪ | |  |  |
| 105 | **የትምህርት ሁኔታ?** | **1**. ያልተማረ  2. መጻፍና ማንበብ ብቻ  3. የመጀመሪያ ደረጃ ት/ትያጠናቀቀ  4.ሁለተኛደረጃ ት/ት ያጠናቀቀ  5. የኮሌጅ/ ዩኒቨርሲቲ | |  |  |
| 106 | **በአማካይ የቤተሰቦ ወርሃዊ ገቢ ስንት ነው?** | **1. ≤500**  **2. 501-1000**  **3. 1001-1500**  **4. 1501-2000**  **5. >2000** | |  |  |
| **ክፍል II: ከጤና ጋር የተያያዙ መረጃዎች** | | | | | |
| 201 | }pTÖ< KU” Á¡M Ñ>²? q¾wI/i | _______ቀን | |  |  |
| 202 | }pTÖ< U” ›Ã’ƒ ’¨<; | 1 ¨<HT ðdi  2. SÓM ¾}kLkKuƒ  3 ÅU ¾}kLkK  4. ÅU“ SÓM | |  |  |
| 203 | **አች.አይ. ቪ ቫይረስ በደምዎ ይገኛል;** | 1. **አዎ**  **2. የለም** | |  |  |
| 204 | }.l 205 ›**ዎ** ŸJ’ ¾CD4 SÖ”I/i e”ƒ ’¨< ? | _______ሴሎች በማይ ክሮሊትር | |  |  |
| ክፍል: III ›ÒLß G<’@ታ©‹ | | | | | |
| 301 | KSÖØ ¾UƒÖkS<ƒ ¨<H Ÿ¾ƒ ’¨< ¾U}Ñ–<ƒ; | | 1 ¾ÓM ቧ”ቧ  2. ¾I´w ቧ”ቧ  3 ¾¨”´ ¨<H  4. ¾Ñ<ÉÔÉ ¨<H |  |  |
| 302 | ¾ÓL‹G< i”ƒ u?ƒ ›L‹G<; | | 1 ›**ዎ**  2. ¾K’U |  |  |
| 303 | }.l 302 ¾K’U ŸJ’ i”ƒu?ƒ ¾ƒ ’¨< ¾UƒçÇÆƒ; | | 1 ¾I´w i”ƒ u?ƒ  2. T@Ç LÃ  3. ¨”´ |  |  |
| 304 | UÓw ŸSSÑv‹G< uòƒ °Í‹G<” ƒታÖvL‹G<; | | ›**ዎ**  ›”}ÖwU |  |  |
| 305 | }.l 302 ›¨ ŸJ’ uUÉ” ’¨< ¾UƒታÖu<ƒ; | | udS<“  ÁKdS<“ |  |  |
