# Supplementary material for: Prevalence of enteric pathogens, intestinal parasites and resistance profile of bacterial isolates among HIV infected and non-infected diarrheic patients in Dessie Town, Northeast Ethiopia
Source: PLoS One. 2020 Dec 15;15(12):e0243479. doi: 10.1371/journal.pone.0243479 (PMC7737993; doi:10.1371/journal.pone.0243479)
Supplement: Attachment — Submitted filename: Response to Reviewers.docx [file pone.0243479.s002.docx]

**Author’s Response to Editor and Reviewer comments**

**Manuscript ID number:**
PONE-D-20-27382

**Title of paper:**
Prevalence of Enteric Pathogens, Intestinal Parasites and High Resistance rate of Bacterial Isolates to Commonly Prescribed Antibiotics in HIV Infected and Non-Infected Diarrheic Patients from selected Health Facilities in Dessie town, Northeast Ethiopia

**General**

We thank all the reviewers for critically reviewing our manuscript which helps us to give better clarity to the paper and make it scientifically strong. We thank the PLOS ONE academic editors for their valuable comments and for giving us an opportunity to revise the manuscript.

All the questions raised by the Reviewers and Editor have been addressed; and the manuscript is modified accordingly. Moreover, the requested Editorial corrections are addressed in both the revised manuscript and the response letter. Changes are shown with track changes in the file labeled ‘Revised Manuscript with Track Changes’. The point by point response is given below.

**Editor Comments:**

1. Please ensure that your manuscript meets PLOS ONE's style requirements, including those for file naming.

***Author’s Response:***

- **Thanks for the significant editorial comment. The revised manuscript is now updated and meets PLOS ONE's style requirements, including those for file naming.**

2. Please include additional information regarding the survey or questionnaire used in the study and ensure that you have provided sufficient details that others could replicate the analyses. For instance, if you developed a questionnaire as part of this study and it is not under a copyright more restrictive than CC-BY, please include a copy, in both the original language and English, as Supporting Information.
   ***Author’s Response:***

- **Thank you for the comment. Copy of the questionnaire in both the original language and English is attached as Supporting Information.**

3. We note that you have provided funding information that is not currently declared in your Funding Statement. However, funding information should not appear in the Acknowledgments section or other areas of your manuscript. We will only publish funding information present in the Funding Statement section of the online submission form. Please remove any funding-related text from the manuscript and let us know how you would like to update your Funding Statement. Currently, your Funding Statement reads as follows: [The author(s) received no specific funding for this work.]

***Author’s Response:***

- **Thank you and corrections are made as per the comment; the Funding Statement is amended and written as: “The authors received no specific funding for this work.”**

**Moreover, the amended statement is included within the cover letter.**

4. Please ensure that you have an ORCID iD and that it is validated in Editorial Manager.

***Author’s Response****:*

- **Thank you for the valuable comment. Based up on the comment, pre-existing ORCID iD of the corresponding author is authenticated in the PLOS ONE Editorial Manager.**

5. We note that you have indicated that data from this study are available upon request. PLOS only allows data to be available upon request if there are legal or ethical restrictions on sharing data publicly.

***Author’s Response****:*

- **Thanks for the comment. Since the study included HIV positive patients, ethical restrictions do not allow us to share their data. However, the manuscript contains all the necessary information.**

6. Please upload a copy of Supporting Information S1 Questionnaire. which you refer to in your text on page 29.

***Author’s Response****:*

- **Thanks, Supporting Information S1 Questionnaire is uploaded with the revised manuscript.**

7. We noticed you have some minor occurrence of overlapping text with the following previous publication(s), which needs to be addressed.

***Author’s Response****:*

- **Thanks, comment well taken; we have tried to rephrase overlapping and duplicated texts as per the comments.**

**Reviewer #1 comments**

1. The authors need to discuss the recommended care of salmonella and shigella (both Ethiopian guidelines and WHO guidelines). For example, current WHO Shigella recommendation are to treat with Cipro. Your study suggest that guideline would work well in this region, which is reassuring. However, you then suggest Cipro as an “alternative” treatment, when in fact perhaps it should be the first line (as per guidelines)? I would suggest including the current treatment recommendation in the introduction and discussing how your results should inform these recommendations in the discussion.

***Author’s Response:***

- Thanks for the invaluable comment. We addressed this in both sections [Ref 21-23]

2. I’m not sure that your conclusion that “the prevalence of these pathogens are high” (paraphrased) is valid. You only recruited children with diarrhea, so you were always going to see a high prevalence of enterics pathogens, and your discuss of other studies suggest that your prevalence is the same or lower than other studies have observed.

***Author’s Response:***

- **Thank you for the substantial comment. We have revised as per the comme****nt.**

3. The discussion focuses heavily on comparing your results with other work. While this is somewhat necessary for a discussion, many of the paragraphs do not appear to have an overarching point to the comparison. The discussion would be greatly improved by the authors make it clear to the reader what they should take away from each comparison. For example, in the second paragraph you compare prevalences of Shigella to other studies, what is your conclusion from this comparison?

***Author’s Response:***

- **Comment well taken and after each comparison a concluding sentence is added, we thank you very much for this.**

4. In the statistical methods it would be better to identify which methods are answering which questions, i.e. you used descriptive methods to estimate the prevalences, and then model to associate exposures with the presence of pathogens. This would make it crystal clear to the reader what you are going to do.

***Author’s Response:***

- **Thank you for the valuable comment. The statistical methods part is amended accordingly.**

5. You do not mention that you’re going to do a corelates of pathogen infection analysis in the introduction, you only mention looking at the prevalence. I was surprised by your correlates of infection analysis when I reached it in the results. Making this a clear goal of the paper in the introduction would be helpful.

***Author’s Response:***

- **Thanks, and we have added a clear statement stating about the goal of the paper to do corelates of pathogen infection analysis and assess possible associated risk factors at the end of the introduction part of the revised manuscript.**

6. There are some minor grammar errors, although the paper is generally well written:
   Background, first paragraph, third line: “safe water is limited”
   Background, fourth paragraph, seventh line: “5 times more bacteraemia”
   Methods, first para, second line, “and two randomly”
   Conclusion, fourth line, “On the other hand”

***Author’s Response:***

- **Comments well taken and the manuscript corrected accordingly, thanks.**

7. In the methods the sentence beginning “ A total of 354 HIV infected…” should be moved to the results.
   ***Author’s Response:***

- **Thanks; we really appreciate the comment which helped us to notice a gap. Since the result section has the similar information, we rather felt the data collection section lacks about how the data was collected. Hence, we revised the sentence and included it under this section to make the reader aware from the beginning that data was collected from the study participants conveniently.**

8. In the antimicrobial susceptibility testing paragraph in the methods, you define the abbreviation CLSI two times.

***Author’s Response:***

- **Comment addressed in the method section, thanks.**

**Finally we thank you for your critical review of the paper.**

Sincerely,

Aster Tsegaye, PhD
